# Correlations between Community Structure and Link Formation in Complex Networks

**DOI:** 10.1371/journal.pone.0072908

**Published:** 2013-09-06

**Authors:** Zhen Liu, Jia-Lin He, Komal Kapoor, Jaideep Srivastava

**Affiliations:** 1 Web Sciences Center, School of Computer Science and Engineering, University of Electronic Science and Technology of China, ChengDu, SiChuan, China; 2 Department of Computer Science and Engineering, University of Minnesota, Minneapolis, Minnesota, United States of America; Semmelweis University, Hungary

## Abstract

**Background:**

Links in complex networks commonly represent specific ties between pairs of nodes, such as protein-protein interactions in biological networks or friendships in social networks. However, understanding the mechanism of link formation in complex networks is a long standing challenge for network analysis and data mining.

**Methodology/Principal Findings:**

Links in complex networks have a tendency to cluster locally and form so-called communities. This widely existed phenomenon reflects some underlying mechanism of link formation. To study the correlations between community structure and link formation, we present a general computational framework including a theory for network partitioning and link probability estimation. Our approach enables us to accurately identify missing links in partially observed networks in an efficient way. The links having high connection likelihoods in the communities reveal that links are formed preferentially to create cliques and accordingly promote the clustering level of the communities. The experimental results verify that such a mechanism can be well captured by our approach.

**Conclusions/Significance:**

Our findings provide a new insight into understanding how links are created in the communities. The computational framework opens a wide range of possibilities to develop new approaches and applications, such as community detection and missing link prediction.

## Introduction

Recently, study of link formation has attracted much attention from disparate scientific communities. This is because understanding the mechanism of link formation can help us predict links occurring in the future accurately and, in turn, accurate link prediction indeed gives evidence to some underlying mechanisms that drive network evolution [1], [2]. Researchers have proposed a variety of link prediction methods from different perspectives of network dynamics [3]–[7]. In some real-world networks, nodes which share similar properties or attributes tend to create links to each other and cluster together. For example, persons in a social network who have similar age, location and hobbies are very likely to be friends, and researchers who have similar research interests possibly work together on a paper in a co-authorship network. Such a phenomenon is also called homophily [8]. By investigating these shared attributes, some algorithms have applied classical machine learning methods to predict latent links in the networks [3]–[5]. However, such type of information, also known as domain knowledge, may not be easy to obtain in real life due to concerns associated with confidential data protection or personal privacy preservation. To overcome such restraints, some simple but effective methods have been proposed by utilizing the local clustering features derived using only the topological information of the network, such as the methods of common neighbors, Jaccard, Adamic Adar [6], resource allocation [7], and CAR-based indices [9] etc. Despite the theoretical and practical advances made on network evolution and link prediction in recent years, the mechanism of link formation is still vague because the process of link formation may be a result of the joint influence of several mechanisms such as small world effect [10], preferential attachment [11], rich-club [12], etc.

Usually, a small world network is described as such a network in which geodesic distance between pairs of nodes is small relative to the total number of nodes in the network. The small world network generated by the well-known Watts-Strogatz model has relatively high clustering. This indicates that clustering might be one of the essential factors that ensures short communication paths for every two nodes in the small world network. The scale-free network generated by Barabási-Albert (BA) model has shown that nodes prefer to connect to nodes that already have many links and this process is known as preferential attachment [13]. For example, on the World Wide Web we are more familiar with the highly connected web pages like portals, and therefore are more likely to link to them. The process of preferential attachment will promote the formation of few hub nodes who own the majority of the network links. As a result, the scale-free network will generally exhibit higher clustering than the random network. Rich-club phenomenon exhibits another important mechanism of link formation. It has been validated empirically that in Internet network, some hub nodes, like backbone routers, tend to connect together to form a rich-club [12]. The rich-club can improve the efficiency of traffic routing and provide the capacity to resist node attacks and prevent the network from breaking down easily. Recently, graphlet-based edge clustering also reveals pathogen-interacting proteins [14]. All the above discussed mechanisms imply that clustering is an ubiquitous characteristic for the evolution of complex networks. Previous studies have also verified that local clustering measures, such as the nodes clustering coefficient, can be utilized to improve the accuracy of link prediction, yet they did not give strong justifications for their methods [15].

Due to the importance of clustering in the complex networks, we must explore the process of link clustering in the networks carefully. Since links tend to cluster in the communities, it provides researchers with the possibility to explore any underlying correlations between network community structure and link formation. Cannistraci et al. [9] and Yan et al. [16] had noticed the significance of community structure and proposed community-related link prediction approaches respectively. For example, Cannistraci et al. proposed a new paradigm to support link formation called local community paradigm (LCP), which emphasizes the role of the local network community structure in link formation. Their previous works have given us good hints to further study on this problem. In this article, by studying the theory of network partitioning, we present a novel network partitioning algorithm called Fast probability Block Model (FBM) which is based on the greedy strategy. We assess the performance of our algorithm by applying it to the problem of missing link prediction on various real-world networks. Experimental results show that our algorithm improves both prediction accuracy and computational efficiency compared with conventional methods. Meanwhile, by analyzing links having high connection likelihoods in the communities, we find that these links tend to cluster and form cliques. We therefore conclude three principles to demonstrate such mechanism of link formation. The experimental results verify empirically that the mechanism can be well captured by our approach.

## Materials and Methods

### Theory for Network Partitioning and Link Probability Estimation

Communities, which are also called modules or clusters, exist widely in real-world networks. For social networks, a community could be a group of people with common interest or location. For biological networks, a community could be a group of cells or proteins with common function. From the perspective of graph theory, a complex network is usually treated as a graph and accordingly a community is demonstrated as a subgraph having dense links. Community detection is a fundamental task to exploit the subgraphs or blocks with different properties and functions nested in a network (In the latter part of this article, we call a subgraph as a block). Researchers have proposed a variety of community detection approaches in recent decades [17]–[21], [21]–[25]. However, current approaches are “biased” for they are often related to some complex structural features such as sparsity, heavy-tailed degree distribution and short diameter, etc., and also strongly depend on the specific application [26]–[28]. As a result, so far, there is no such a universal measure which can allow an unbiased determination of whether a block obtained in a given network is a true community or not. This also suggests that different community detection measures have different merits such that researchers have the flexibility to choose a suitable measure to study a specific community detection problem. In this article, in order to study the correlations between link formation and community structure effectively, we adopt the measures of inner link density and connecting link density to quantitatively ascertain a community. If a block 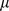 has 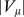 number of nodes and 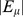 number of edges, the inner link density of the block is defined as
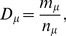
(1)where 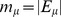 and 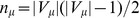. According to Eq. (1), the inner link density 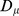 denotes the ratio of the actual number of inner links to the maximum possible number of links in the block 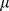. Notice that, when 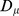 equals 1, the block will reach the highest density and form a complete subgraph which is also called a clique. To quantitatively describe the link density between two blocks, we also define the connecting link density between every two blocks 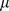 and 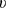 in a given network as follows. Suppose 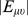 is the number of edges between two blocks, while 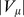 and 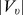 are the number of nodes in the block 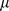 and block 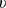 respectively, the product of 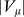 and 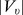 denotes the maximum number of links that can exist between the two blocks. So the connecting link density is defined as
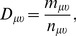
(2)where 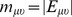 and 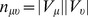. Here, we define a community as such a block in a given network which has relatively high inner link density and relatively low connecting link density with other blocks. We note that the structure and characteristics of the communities naturally exhibit a statistical mechanism in which links are more likely to form within the community, whereas they are less likely to be established between communities. If a network could be partitioned into communities properly, we will likely be able to estimate the probability of link formation between any pair of nodes based on the distribution of communities.

**Figure 1:**
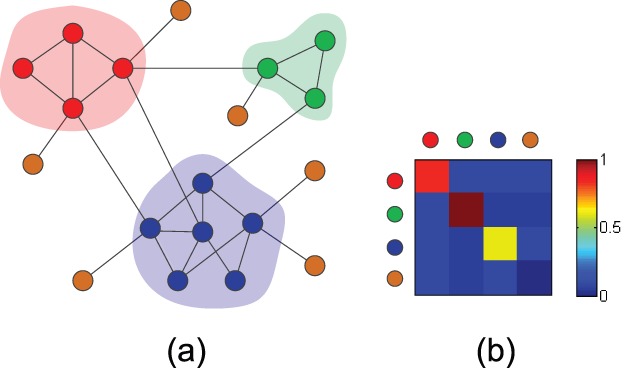
An example network illustrating the relationship between community distribution and link density matrix. (a) The community distribution of the network. (b) The link density matrix of the corresponding community distribution.

In Fig. 1(a), we give an example of a network which has been partitioned into three possible communities in accordance with our community definition, which are marked with different colors. The conventional community detection methods tend to enforce a binding of every node in the network to some particular communities. But, by doing so, some nodes with small degree, like leaf nodes, are introduced into the community as noise to decrease the inner link density of the community, and this possibly result in the so-called issue of the resolution limit for community detection [29]. We think this is particularly true in scale-free networks since there are a large number of small degree nodes in such a kind of network. Unlike the conventional point of view for community detection, we consider that these nodes do not belong to any communities and should be categorized as a special “community”. Therefore, the leaf nodes, which are marked with brown color in Fig. 1(a), are grouped together as a special “community” which has no inner links. Of course, in this “community”, links have a very small chance of being established between pairs of nodes. If we partition a network into communities in this manner, we can obtain a link density distribution matrix by using Eq. (1) and Eq. (2) to calculate the link densities within and between communities. In Fig. 1(a), we partition the network into four communities including a special “community”, thereafter we obtain a link density matrix shown in Fig. 1(b).

One network partition can only provide one link density distribution while there usually exist many possible network partitions. If we want to estimate the link probabilities for any node pairs in a given network, based on the theory of statistics, we need to obtain as many independent network partitions as possible by doing multiple rounds of network partitioning. Such a procedure is also known as sampling. Considering that an observed network (a network having missing links) has an adjacency matrix 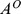, we use a network partitioning method 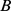 to partition the observed network into communities. According to the Bayes theorem, the link probability of a node pair 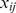 can be estimated as
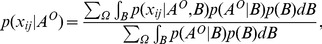
(3)where 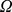 denotes the space of samplings. For a node pair 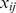 within a community 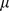, we suppose that 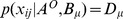. Due to the high inner link density existing in the community 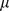, it follows that 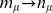. We obtain



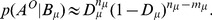
(4)Let 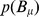 equal a constant. Eq. (3) can be rewritten as
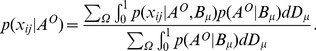
(5)


Using Eq. (4) and Eq. (5), one can obtain
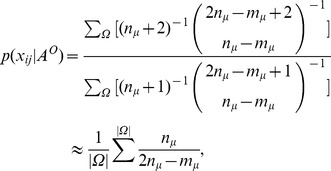
(6)where 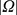 denotes the times of the sampling. Likewise, for a node pair 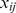, where node 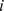 is in community 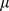 and node 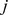 is in community 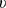, we suppose that 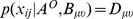. Due to the low connecting link density between the community 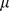 and community 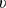, it follows that 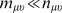. We obtain



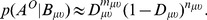
(7)Let 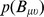 equal a constant. Eq. (3) can be rewritten as
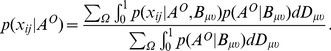
(8)


Using Eq. (7) and Eq. (8), one can obtain
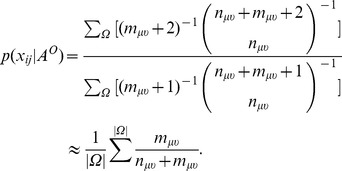
(9)


In the **Results** section, by applying the network partitioning algorithm FBM proposed in the next section, we mainly use Eq. (6) and Eq. (9) to estimate link probabilities for missing links and non-existent links, namely the connection likelihood for those node pairs having no links in the observed network. Notice that, for the special “community” introduced in this section, the link probabilities for those node pairs within it are always zero.

### Algorithm of Fast Block Probabilistic Model

According to the theory of network partitioning, our goal is to partition the network into a set of blocks and ensure that each block is either a community or a special community composed of some small degree nodes. It’s not trivial to find all the communities in a given network by an exhaustive search because the search space is usually very large. To perform this task efficiently, we proposed a Fast Block probabilistic Model (FBM) by applying the greedy strategy (A greedy algorithm is an algorithm that follows the problem solving heuristic of making the locally optimal choice at each stage with the hope of finding a global optimum. In some cases, a greedy strategy does not in general produce an optimal solution, but nonetheless a greedy heuristic may yield locally optimal solutions that approximate a global optimal solution in a reasonable time). Compared with conventional methods using the rule of Metropolis-Hasting [30], our algorithm can obtain huge improvement in computational efficiency in accordance with the experimental results shown in the **Results** section. The FBM algorithm can be described as follows:

**Input:** a network 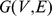;

**Output:** an array of communities 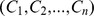 of the network 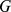;

**1
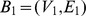
** and 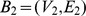;//**Randomly partition the network 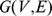 into two blocks 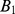 and 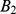. Let 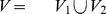, 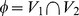 and 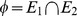.**

**2** **for** *each* 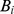 **do**

**3** 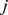 = 1;

**4 while** 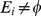 **do**

**5** 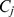 = CommunityFind(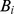);//**Find a community 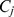 with relatively high inner link density in the 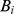.**

**6** Output(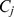);

**7** Remove(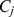, 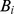);//**Remove 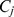 from 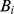 including nodes and edges which belong to 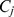 and links between 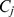 and 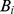.**

**8** 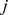 = 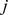
+1

**9 end**

**10** Output(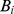);/**/Obtain a special community composed of a set of small degree nodes.**

**11 end**

To find a community in step 5, we need to determine whether a block is a community by calculating its link density. The procedure of finding a community is described as follows:

**Input :** a block 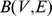;

**Output:** a community 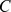;

**1
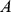
** = Density(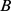);//**Calculate the inner link density of the block 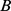 by using Eq. (1)**

**2** **while** 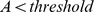 **do**//**A** 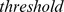 for link density is an accepted maximum value set in advance. 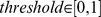.

**3** Sort(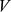);//**Sort all nodes by degree in descending order.**

**4** 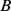 = Remove(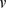,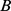);//**Pick out the first node 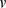 owning the least degree in the ordered node list. Remove node 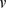 and edges connecting between 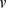 and 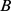.**

**5** 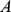 = Density (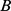);//**Recalculate the inner link density of block 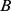.**

**6 end**

**7** 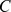 = 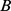;

**8** Output(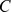);

We don’t need to specially provide a mechanism in our algorithm to ensure a relatively low connecting link density between blocks due to the fact that most real-world networks are sparse [31]. By implementing our algorithm, we find that it can keep the number of links between every two blocks low, automatically; i.e., every two blocks has relatively low connecting link density. In fact, we have also verified that our algorithm can still work well even through the network is relatively dense. For a single running of the algorithm, we can obtain one network partition. According to the sampling theory described in the former section, we need to implement the algorithm iteratively to obtain enough independent network partitions. To ensure that each network partition is independent, we have adopted a simple but effective trick in which we partition the network into two blocks randomly in the first step in our algorithm. Unlike the rule of Metropolis-Hasting which requires numerous node moves to ensure that the next partition is independent of the former one, the first step can ensure that our algorithm achieves this goal efficiently. This is also a reason that our algorithm is much faster than the conventional ones, such as HRG [32] and SBM [33]. As a result of the first step, a given network partition obtained by our algorithm always contains two special communities which are composed of small degree nodes. On the other hand, if we remove this step, our algorithm will degenerate to a pure community detection algorithm.

**Figure 2:**
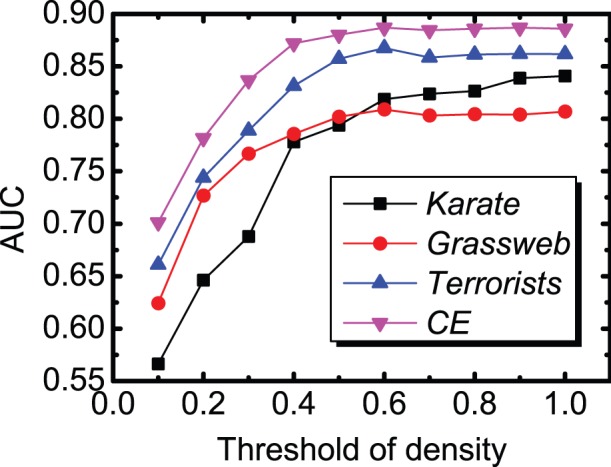
The dependence of AUCs on the thresholds of link density for the four networks.

Before using the FBM algorithm to implement link prediction, we still need to determine an uncertain or free parameter. As stated in the algorithm, the 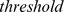 for link density must be chosen carefully (the 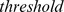 is an accepted maximum value of the link density to identify a community in our algorithm). We try to pick out the optimized value of the 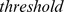 by observing the variations in prediction accuracy (see the definition of accuracy measure in the **Results** section) with different link density settings. The fraction of missing links is set to ten percent for four small-sized networks which we are about to use to test in the **Results** section (We obtained similar results on using different fractions of missing links) and the accuracy variation curves plotted for the four networks are shown in Fig. 2. We find that the accuracy of link prediction tends to converge after the link density is larger than 0.5 and reaches the best when the 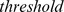 for link density is set to 1 such that a block corresponds to a clique. Therefore, we set the 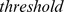 to 1 while implementing the networks partitioning for all testing networks in this article. Meanwhile, since we’ve validated that FBM approach has the best accuracy performance when the threshold is equal to 1, it turns out that the procedure of finding a community is equivalent to the procedure of finding a maximum clique in a given network. According to our experiments, we observe that, by using a fast maximum clique detection algorithm [34] to replace the procedure of finding a community in our algorithm, the FBM approach can provide same accurate results but perform even faster (the source code of the FBM algorithm is freely available upon request).

### Data Description

In this article, we consider eight real-world networks for evaluation. (1) Karate: Social network of friendships between 34 members of a karate club at a US university in the 1970s [35]. (2) Grassweb: Food web of a grassland ecosystem, i.e., a network of predator-prey interactions between species [36].(3) Terrorists: A network of associations between terrorists [37]. (4) C. elegans (CE): The neural network of the nematode worm C. elegans, in which an edge joins two neurons if they are connected by either a synapse or a gap junction [38]. (5) Political Blogosphere (PB): Political blogosphere is compiled by Lada Adamic and Natalie Glance. Links between blogs were automatically extracted from a crawl of the front page of the blog [39]. (6) Online Dictionary of Library and Information Science (ODLIS): ODLIS is designed to be a hypertext reference resource for library and information science professionals, university students and faculty, and users of all types of libraries [40]. (7) PPI net1: A yeast protein-protein interaction network. Some links in this network are not reliable. This is the same PPI used as network1 in Cannistraci et al. [9]. (8) PPI net2: A yeast protein-protein interaction network. Some links in this network are not reliable. This is the same PPI used as network2 in Cannistraci et al. [9]. Here, we only consider the giant connected component and every network is treated as an undirected network. The statistics on the topological features of the eight networks are summarized in Table 1.

**Table 1:** Statistics on the topological features of the eight networks.

	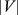	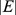	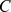	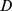	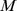	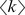	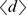
Karate	34	78	0.588	0.139	0.416	4.588	2.408
Foodweb	75	113	0.497	0.041	0.635	3.013	3.875
Terrorists	62	152	0.58	0.08	0.529	4.903	2.508
CE	297	2148	0.308	0.049	0.397	14.465	2.946
PB	1222	16714	0.36	0.022	0.426	27.355	2.738
Odlis	2898	16376	0.351	0.004	0.456	11.302	3.17
PPI-1	4036	10411	0.094	0.001	0.576	5.159	4.412
PPI-2	4385	12234	0.128	0.001	0.569	5.58	4.424

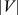
 and 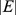 denote the number of nodes and links. 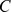 and 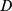 are the average clustering coefficient and the density of the network, respectively. If a vertex 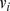 has 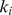 neighbours, 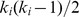 edges could exist among the vertices within the neighbourhood. Thus, the local clustering coefficient for a network can be defined as 
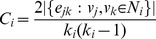
 [39], where 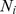 denotes the neighbours of 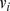. 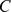 is defined as 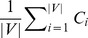. 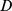 is defined as 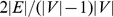. 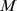 is the modularity of the network defined as 
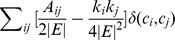
 [26], where 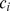 denotes a community which includes the vertice 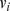 and 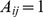 if vertices 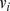 and 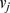 are connected and 0 otherwise and 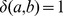 if 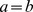 and 0 otherwise. 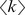 and 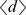 denote the average degree and the average shortest distance.

## Results

To estimate the connection likelihood of missing links, researchers have developed various probabilistic prediction models in recent years. A typical model, Hierarchical Random Graph (HRG), which was proposed by Aaron Clauset et al., was applied to predict missing links in some networks with obvious hierarchical structure [32]. Based on a similar principle of statistics as used by HRG model but from another angle of view, Roger Guimerà et al. proposed a Stochastic Block Model (SBM) which can predict both missing links and spurious links and is able to give much better accuracies of prediction on various kinds of networks than current popular methods including the HRG approach [33].

Because the SBM algorithm is a state-of-the-art approach which has very outstanding accuracy performance of link prediction on undirected networks without additional node’s or edge’s attribute information, we mainly make performance comparisons on both accuracy of missing link prediction and computational efficiency between our algorithm and the SBM approach. The measures for prediction accuracy we used here are AUC (area under the receiver operating characteristic curve) and precision. The AUC is a widely used accuracy measure which can be interpreted as the probability that a randomly chosen missing link is given a higher score than a randomly chosen non-existent link. The precision is also a popular measure which can be defined as the proportion of top-ranked candidate links matched to the actual missing links. The prediction accuracies of the common neighbors method are presented here as a baseline. Our algorithm and the SBM approach have a common characteristic which is that in both the approaches one is required to sample network partitions. To ensure that the comparison is fair, we apply the same sampling standard to both the approaches which is set to 50 times (This sampling standard is validated empirically by our experiments which can ensure that both the approaches obtain stable prediction accuracy on the testing networks and more samplings only incur more time consumption for the implementation). The machine we use for testing is a desktop with a processor of Intel (R) Core (TM) i7 CPU 930 @ 2.8 Ghz and 8 Gigabytes memory.

**Figure 3:**
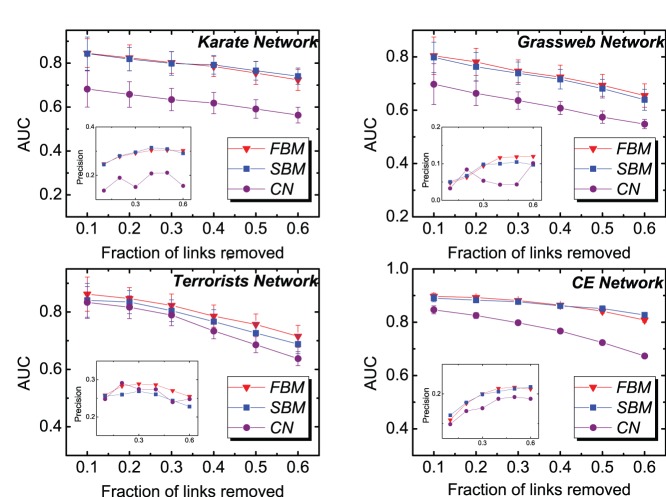
Accuracy comparisons for missing link prediction between FBM, SBM and CN approaches on four networks. Each of AUC value and precision value is averaged over 100 implementations and the error bar represents the standard deviation.

**Figure 4:**
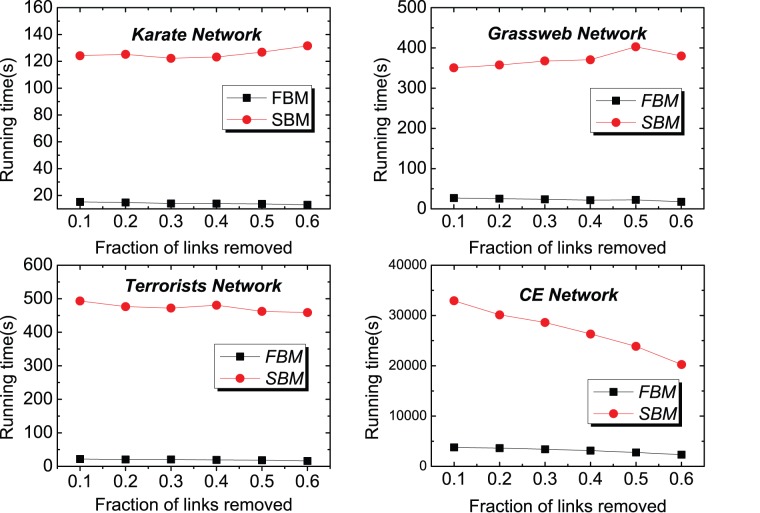
Comparisons of computational efficiency on four networks. Each value of running time is the cumulative time for 100 implementations.

Here, we use four real-world networks including Karate, Grass-web, Terrorists and CE for evaluation. The links removed from the network constitute the probe set of missing links while the rest of the network constitutes the training set. The prediction accuracies on the four networks, measured by AUC and precision, are plotted in Fig. 3 and the corresponding comparisons for running time (the unit is seconds) are shown in Fig. 4. To ensure that the results can be trusted, each value of the accuracy is obtained by averaging over 100 implementations with independently random network divisions of training set and probe set while the error bars denote the standard deviation. Accordingly, each value of the running time is the cumulative time over 100 implementations.

According to the AUC comparison results shown in Fig. 3, the FBM approach performs better than the SBM approach on the networks of Grassweb and Terrorists, and has very close accuracy results to that of the SBM approach for the other two networks. As for the precision measure, FBM approach also obtains similar results compared with SBM approach. Meanwhile, according to the computational efficiency comparison results shown in Fig. 4, the running time used by the FBM approach is far less than that used by the SBM approach. During the experiments, we observed that the running time consumed by the SBM approach increased rapidly along with the size of the network while that of the FBM approach increased gradually, and this indicates that the SBM algorithm has much higher time complexity than the FBM algorithm and is also the main reason that we only choose four networks with relatively small size for making performance comparison. We have also performed accuracy and running time comparisons with some other popular local similarity indices, such as classical local similarity indices and CAR-based indices [9] on six networks including two large-sized networks (see Figure S1, Table S1, Table S2 and Table S3 in Appendix SI for detailed results). All the experimental results confirm that the FBM approach is able to provide very good accuracy for missing link prediction on real-world networks with superior computational efficiency.

## Analysis

### Link Prediction within Network Communities

**Figure 5:**
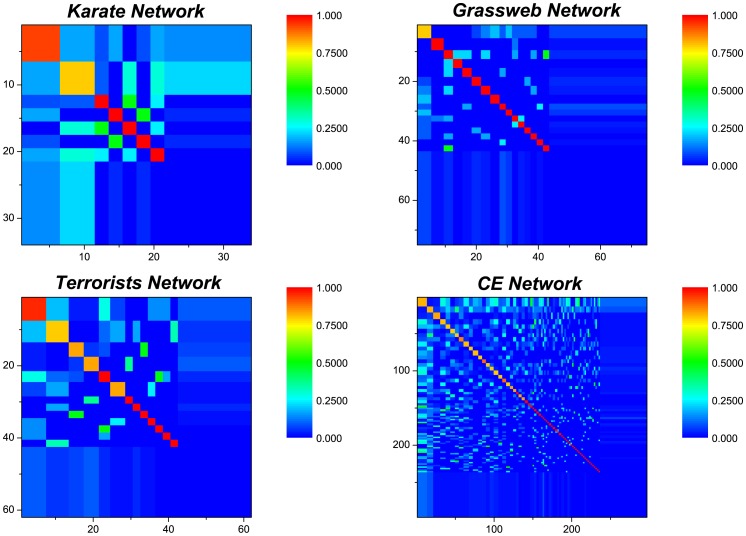
Link density distributions for the four networks. The diagonal highlighted blocks are corresponding to communities with link densities which are not less than 0.8.

**Figure 6:**
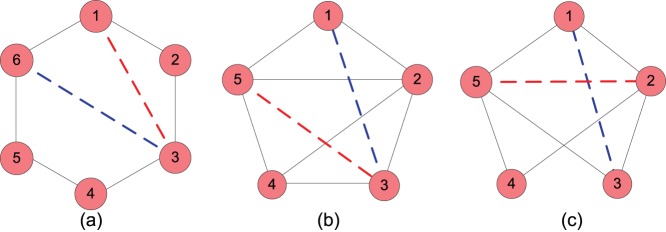
Three artificial networks illustrating the link formation mechanism in the communities.

In this section, we try to find out why the FBM approach can give very good accuracy for missing link prediction on the tested networks. Using the FBM approach, we partition the networks into communities and obtain link density distributions shown in Fig. 5. We note that some node pairs within the communities have a relatively high connection likelihood. This implies that these node pairs are possible to form links within the communities. By further analyzing the local structures connecting to these node pairs, we deduce that there are mainly three important principles driving link formation in the communities. To demonstrate the three principles easily, we give three typical cases shown in Fig. 6, each of which is able to uncover one kind of links which are likely to form in the communities.

Fig. 6(a) shows a ring structure with six nodes labelled by numbers and two possible links for node pair (1,3) and node pair (3,6) which are denoted by red dashed line and blue dashed line. We evaluate the likelihoods of the two possible connections by applying the FBM approach to the network. The results show that the link probability of node pair (3,6) is only 50 percent of that of node pair (1,3) which means that the node pair (1,3) is more likely to connect together compared with node pair (3,6). We note that if a link is added to node pair (1,3), a clique (1,2,3) will be established. This indicates that a link tends to establish a clique in a network. Fig. 6(b) shows a five-node network and two possible links for node pair (1,3) and node pair (3,5) which are also denoted by blue dashed line and red dashed line, respectively. After calculating the link probabilities of the two node pairs, we find that the link probability of node pair (1,3) is 75 percent of that of node pair (3,5). The difference between the two link probabilities can be accounted from the observation that an addition of link (3,5) will form a larger clique (2,3,4,5) than the clique (1,2,3) if link (1,3) is added. This case demonstrates that a link tends to create a larger clique first in a network when there are many options available to choose from. Fig. 6(c) shows another interesting phenomenon of link formation. After calculation, the link probability of node pair (2,5) is 1.5 times higher than that of node pair (1,3). We find that link (2,5) can create three cliques including (1,2,5), (2,3,5) and (2,4,5) while link (1,3) is only able to create two cliques, i.e., (1,2,3) and (1,3,5). This result implies that if adding a link is able to create more cliques, the link will have higher likelihood of being established. Based on the three typical cases, we summarize three principles to explain how a link is created in a given community.

- Links tend to be established such that they form a clique in a given community.
- A link prefers to create a large-sized clique rather than a small-sized clique in a given community.
- A link tends to form as many cliques as possible in a given community.

**Figure 7:**
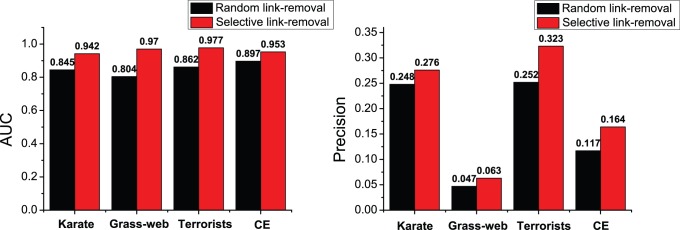
Accuracy comparisons between predicting links selectively removed from the communities by following the three link formation principles and predicting links removed randomly. The fraction of links removed is always 10 percent.

The three principles reveal that links tend to cluster and form cliques in the community. To validate whether the link formation mechanism described by the three principles can be well captured by our algorithm, we perform an additional experiment on the four networks. We vary the link density 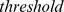 from 0.6 to 0.9 and use the FBM approach to partition each network into communities. In each running, we apply the three principles to selectively remove 10 percent of links which exist in the communities. The set of removed links is treated as the probe set and the rest of the network is treated as the training set. We again use the AUC and precision measures to evaluate the accuracy of prediction and obtain the average results over 100 implementations shown in Fig. 7. As expected, we find that the prediction accuracies for the links removed selectively are much better than those for the links removed randomly. The best AUC result for Karate network is nearly as high as 95% and those for other three networks even exceed 95%. Likewise, the precisions on four networks are improved drastically as well. Even in the worst case of the grassweb network, the precision can achieve 34.04% improvement. The results empirically verify that the FBM approach can indeed identify missing links in the communities very accurately.

### Link Prediction in Noisy Networks

**Figure 8:**
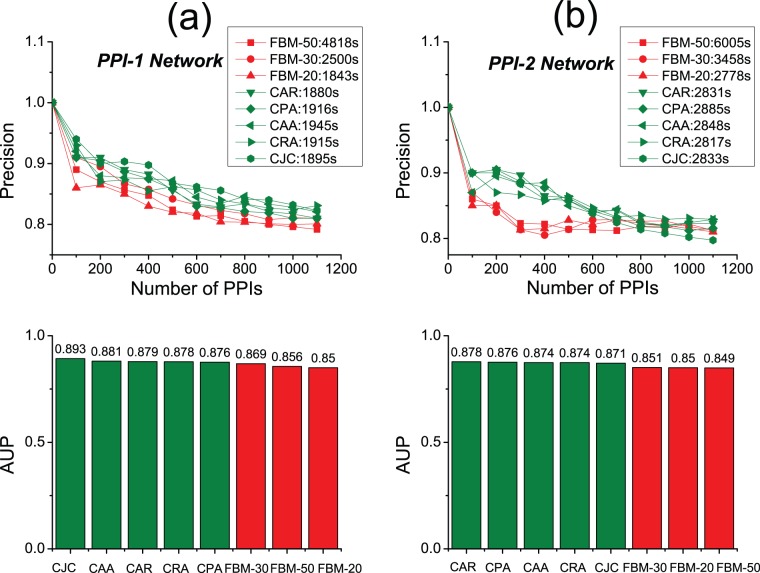
Accuracy and computational time comparisons between FBM and CAR-based approaches on two noisy PPI networks. (a) The upper plot illustrates the precision curves for all approaches on the PPI-1 network while the bars in the lower plot illustrate the corresponding AUP (area under precision curve) values for each approach. (b) The upper plot illustrates the precision curves for all approaches on the PPI-2 network while the bars in the lower plot illustrate the corresponding AUP (area under precision curve) values for each approach. The sampling parameter is tuned by 20, 30 and 50 for the FBM approach. The computational time (seconds) for each method is shown in the legend.

Link prediction in noisy networks (networks having unreliable links) is a vital challenge in the domain of bioinformatics. To further validate the FBM approach’s performance, we assess our approach on two noisy protein-protein interaction (PPI) networks. Because it’s reported that CAR-based indices have outstanding performance of link prediction on the two noisy PPI networks [9], we also adopt the two networks to make comparisons between FBM approach and CAR-based indices shown in Fig. 8. Here, we directly treat each noisy network as the observed network and take an external referee (gene ontology) to validate the accuracy of prediction on the non-adjacent links, and therefore random link-removal is not necessary in this case (for details on this evaluation procedure referring to Cannistraci et al. [9]). The results demonstrate that the FBM approach basically performs worse than CAR-based indices in both precision and computational time. During the experiments, we specially tuned the sampling parameter for the FBM by 20, 30 and 50 respectively. We notice that more or less samplings will both impact the accuracy of the approach negatively (when the sampling parameter is set to 30, it can produce the best accuracy results in terms of the two experiments). And this implies that too many samplings will cause the FBM model over-fitting to the noisy or unreliable network which can’t truly reflect the real link formation mechanism of protein-protein interaction (in other words, unreliable network will mislead the training procedure of the FBM model). Meanwhile, less samplings will inevitably cause the issue of under-fitting. So, the noisy networks have put the FBM into a dilemma and degraded its performance. The results shown in Fig. 8 also verify that CAR-based methods are quite robust to resist noisy information, and this is a merit of CAR-based methods.

## Discussion

In this article, based on the theory for network partitioning and the greedy strategy, we proposed the algorithm of Fast probability Block Model (FBM) which can partition a given network into communities efficiently. By applying it to predict missing links on four real-world networks, the FBM algorithm exhibited slightly better accuracy of prediction and overwhelmingly better computational efficiency than the state-of-the-art method SBM. According to the accuracy comparison results (see Figure S1 of Appendix SI for detailed results), FBM can basically outperform all local similarity indices on six real-world networks. We believe that the FBM approach has the potential to give fairly good accuracy of link prediction on much larger and more complex networks such as massive biological networks, rapidly growing social networks, and World Wide Web networks, etc., for its outstanding performance in prediction accuracy and computational efficiency. Meanwhile, our method merely uses the topological information of the networks. So it is very likely to give enhanced prediction accuracy in specific applications when domain knowledge, such as node properties and edge features, is introduced. However, it must be pointed out that, according to our experimental results, the current version of FBM approach can’t perform very well on the noisy networks for it has the over-fitting limitation to the unreliable network data. And this will be an interesting question for us to explore further in the future.

On the other hand, from the theoretical aspect, we conclude three principles to demonstrate that links are formed preferentially to create cliques in the communities, which shed light on the underlying mechanism of link formation in the communities. In accordance with our experiments, the FBM algorithm can give much better accuracy of prediction for the specific links removed from the communities using the three principles than that for randomly missing links. This result indicates that some rules governing the link formation and clustering in the communities have been well captured by our approach. Previous studies in biological networks have found that, in terms of gene expression, clique represents the most trusted potential for identifying a set of interacting genes [41]. Our work can provide new evidences demonstrating that the process of link formation and clustering in the communities is promoted by the growth of cliques. Furthermore, the mechanism of common neighbors approach, usually explained by the balance theory [42], [43], is actually a special application of the principle (iii). If a given node pair has many common neighbors, it implies that it will form many triangle cliques if a link is added to the node pair. Therefore, the common neighbors approach can still perform well in some cases. But because it only captures one aspect of link formation, it could not give good results of accuracy in our experiments.

## Supporting Information

Figure S1 **Accuracy comparisons for missing link prediction between FBM, CAR-based approaches and classical approaches on six networks.** The fraction of links randomly removed is always 10 percent. Each value of the AUC and precision is averaged over 100 implementations.
(EPS)

Table S1 **Statistics on the average AUCs with standard deviations for the FBM, CAR-based indices and classical indices on six networks with 10 percent of links randomly removed.** Each value of the AUC is averaged over 100 implementations. The values in boldface are the top-3 best results.
(PDF)

Table S2 **Statistics on the average precisions with standard deviations for the FBM, CAR-based indices and classical indices on six networks with 10 percent of links randomly removed.** Each value of the precision is averaged over 100 implementations. The values in boldface are the top-3 best results.
(PDF)

Table S3 **Statistics on the running time (seconds) for the FBM, CAR-based indices and classical indices on six networks with 10 percent of links randomly removed.** Each value of the running time is the cumulative time for 100 implementations. The values in boldface are the best results.
(PDF)

Appendix SI **Appendix to the manuscript.**
(PDF)
