# Supplementary material for: Correlations between Community Structure and Link Formation in Complex Networks
Source: PLoS One. 2013 Sep 6;8(9):e72908. doi: 10.1371/journal.pone.0072908 (PMC3765235; doi:10.1371/journal.pone.0072908)
Supplement: Table S1 — Statistics on the average AUCs with standard deviations for the FBM, CAR-based indices and classical indices on six networks with 10 percent of links randomly removed. Each value of the AUC is averaged over 100 implementations. The values in boldface are the top-3 best results. (PDF) [file pone.0072908.s002.pdf]

Table S1. Statistics on the average AUCs with standard deviations for the FBM, CAR-based indices and classical indices on six networks with 10 percent of links randomly removed.

	Karate	Grassweb	Terrorists	CE	PB	Odlis
FBM	**0.840±0.069**	**0.805±0.062**	**0.860±0.061**	**0.896±0.013**	**0.931±0.001**	**0.919±0.005**
CAR	0.583±0.070	0.514±0.022	0.713±0.056	0.769±0.015	0.892±0.004	0.753±0.008
CPA	0.542±0.136	0.387±0.101	0.610±0.085	0.771±0.020	0.910±0.003	0.853±0.007
CAA	0.586±0.071	0.514±0.022	0.714±0.057	0.771±0.015	0.892±0.003	0.753±0.008
CRA	0.586±0.071	0.514±0.022	0.714±0.057	0.773±0.015	0.894±0.003	0.754±0.008
CJC	0.577±0.067	0.511±0.026	0.712±0.056	0.764±0.015	0.887±0.004	0.749±0.008
CN	0.683±0.082	0.698±0.077	0.833±0.056	0.846±0.014	0.917±0.003	0.891±0.006
PA	0.699±0.111	0.555±0.101	0.667±0.079	0.751±0.018	0.899±0.002	0.862±0.005
AA	**0.719±0.089**	**0.708±0.079**	**0.850±0.059**	**0.863±0.013**	**0.920±0.003**	**0.900±0.006**
RA	**0.729±0.090**	**0.709±0.080**	**0.851±0.060**	**0.867±0.013**	**0.921±0.003**	**0.901±0.006**
JC	0.599±0.068	0.687±0.077	0.823±0.053	0.792±0.012	0.870±0.002	0.833±0.006

Each value of the AUC is averaged over 100 implementations. The values in boldface are the top-3 best results.
