# Supplementary material for: Correlations between Community Structure and Link Formation in Complex Networks
Source: PLoS One. 2013 Sep 6;8(9):e72908. doi: 10.1371/journal.pone.0072908 (PMC3765235; doi:10.1371/journal.pone.0072908)
Supplement: Table S2 — Statistics on the average precisions with standard deviations for the FBM, CAR-based indices and classical indices on six networks with 10 percent of links randomly removed. Each value of the precision is averaged over 100 implementations. The values in boldface are the top-3 best results. (PDF) [file pone.0072908.s003.pdf]

Table S1. Statistics on the average precisions with standard deviations for the FBM, CAR-based indices and classical indices on six networks with 10 percent of links randomly removed.

	Karate	Grassweb	Terrorists	CE	PB	Odlis
FBM	**0.248±0.117**	0.047±0.045	0.259±0.091	**0.120±0.020**	**0.179±0.007**	**0.119±0.008**
CAR	0.149±0.110	0.024±0.040	0.253±0.088	0.092±0.019	0.168±0.007	0.093±0.006
CPA	0.136±0.090	0.040±0.054	0.245±0.089	0.098±0.020	**0.168±0.006**	0.081±0.006
CAA	**0.188±0.105**	0.032±0.043	0.273±0.089	0.100±0.019	0.166±0.007	**0.103±0.007**
CRA	**0.188±0.105**	0.032±0.043	**0.282±0.087**	**0.113±0.019**	**0.171±0.007**	**0.118±0.072**
CJC	0.094±0.087	0.027±0.046	0.211±0.082	0.071±0.016	0.148±0.009	0.030±0.008
CN	0.138±0.093	0.033±0.052	0.248±0.091	0.100±0.018	0.166±0.007	0.092±0.004
PA	0.076±0.085	**0.054±0.059**	0.117±0.067	0.057±0.015	0.062±0.006	0.033±0.004
AA	0.144±0.099	**0.120±0.081**	**0.289±0.087**	**0.102±0.017**	0.163±0.007	0.099±0.076
RA	0.146±0.103	**0.121±0.082**	**0.289±0.091**	0.101±0.017	0.141±0.006	0.095±0.009
JC	0	0.004±0.026	0.050±0.055	0.024±0.009	0.020±0.003	0.006±0.003

Each value of the precision is averaged over 100 implementations. The values in boldface are the top-3 best results.
