# Supplementary material for: Correlations between Community Structure and Link Formation in Complex Networks
Source: PLoS One. 2013 Sep 6;8(9):e72908. doi: 10.1371/journal.pone.0072908 (PMC3765235; doi:10.1371/journal.pone.0072908)
Supplement: Table S3 — Statistics on the running time (seconds) for the FBM, CAR-based indices and classical indices on six networks with 10 percent of links randomly removed. Each value of the running time is the cumulative time for 100 implementations. The values in boldface are the best results. (PDF) [file pone.0072908.s004.pdf]

Table S1. Statistics on the running time (seconds) for the FBM, CAR-based indices and classical indices on six networks with 10 percent of links randomly removed.

	Karate	Grassweb	Terrorists	CE	PB	Odlis
FBM	**6.56**	**14.15**	**13.77**	**260.36**	**12543.63**	98279.09
CAR	11.18	38.89	28.75	685.12	14332.63	62348.42
CPA	10.03	38	28.08	694.60	14175.14	61934.53
CAA	10.07	38.06	29.15	692.94	14283.64	62382.28
CRA	10.10	37.56	28.82	695.26	14428.63	63156.02
CJC	9.87	37.57	28.18	696.38	14494.71	62983.89
CN	8.97	35.21	26.13	669.94	13863.88	**61056.24**
PA	8.78	34.98	26.37	678.94	14012.64	61734.97
AA	9.11	36.25	27.11	684.45	13903.22	61116.24
RA	9.21	36.31	27.18	688.37	14067.76	62159.39
JC	9.02	35.74	26.93	683.93	14122.35	62209.92

Each value of the running time is the cumulative time for 100 implementations. The values in boldface are the best results.
