# Supplementary material for: Correlations between Community Structure and Link Formation in Complex Networks
Source: PLoS One. 2013 Sep 6;8(9):e72908. doi: 10.1371/journal.pone.0072908 (PMC3765235; doi:10.1371/journal.pone.0072908)
Supplement: Appendix SI — Appendix to the manuscript. (PDF) [file pone.0072908.s005.pdf]

Appendix SI: Correlations between Community Structure and Link Formation in Complex Networks

In this article, we also made accuracy comparisons with five local similarity indices and five CAR-based indices on six real-world networks. The local similarity measures including Common Neighbors (CN), Preferential attachment (PA), Adamic-Adar (AA), Resource Allocation (RA) and Jaccard(JC).
(1) The definition of CN index is:

$$s_{xy} = \Gamma_x \cap \Gamma_y, \tag{S1}$$

where Γ_x denotes the set of neighbors of node x.
(2) The definition of PA index is:

$$s_{xy} = \Gamma_x \cdot \Gamma_y, \tag{S2}$$

where Γ_x denotes the set of neighbors of node x.
(3) The definition of AA index is:

$$s_{xy} = \sum_{z \in \Gamma_x \cap \Gamma_y} 1/\log(k(z)), \tag{S3}$$

where $k(z)$ denotes the degree of node z.
(4) The definition of RA index is:

$$s_{xy} = \sum_{z \in \Gamma_x \cap \Gamma_y} 1/k(z). \tag{S4}$$

(5) The definition of JC index is:

$$s_{xy} = \frac{\Gamma_x \cap \Gamma_y}{\Gamma_x \cup \Gamma_y}, \tag{S5}$$

where Γ_x denotes the set of neighbors of node x.

The CAR-based indices are some improved forms of classical indices, including CAR index (improved CN version), CPA (improved PA version), CAA (improved AA version), CRA (improved RA version), CJC (improved JC version). The main idea of CAR-based indices is to use the local community information of two nodes' common-first-neighbours to enhance the prediction capability of classical indices.
(1) The definition of CAR index is:

$$s_{xy} = CN(x,y) \cdot \sum_{z \in \Gamma_x \cap \Gamma_y} \frac{\gamma(z)}{2}, \tag{S6}$$

where Γ_x denotes the set of neighbors of node x. $\gamma(z)$ refers to the sub-set of neighbours of z that are also common neighbours of x and y.
(2) The definition of CPA index is:

$$s_{xy} = e_x e_y + e_x CAR(x,y) + e_y CAR(x,y) + CAR(x,y)^2, \tag{S7}$$

where Γ_x denotes the set of neighbors of node x. e_x refers to the external degree of x, computed considering the neighbours of x that are not common neighbours of x and y.
(3) The definition of CAA index is:

$$s_{xy} = \sum_{z \in \Gamma_x \cap \Gamma_y} \frac{\gamma(z)}{\log(k(z))}, \tag{S8}$$

where $k(z)$ denotes the degree of node z.
(4) The definition of CRA index is:

$$s_{xy} = \sum_{z \in \Gamma_x \cap \Gamma_y} \frac{\gamma(z)}{k(z)}. \quad \text{(S9)}$$

(5) The definition of CJC index is:

$$s_{xy} = \frac{CAR(x,y)}{\Gamma_x \cup \Gamma_y}, \quad \text{(S10)}$$

where Γ_x denotes the set of neighbors of node x.

Upon the tests, we used an improved Matlab program for FBM approach here (Note that, we applied the fast clique detection algorithm in this program as we have stated in the article). Meanwhile, the Matlab program which we used to test here for CAR-based approaches and classical approaches is obtained from the url: https://sites.google.com/site/carlovittoriocannistraci/5-datasets-and-matlab-code/car-based-indices-and-local-community-paradigm.

Fig. S1 shows that the FBM approach outperforms all other indices by AUC measure on all six networks and also outperforms all other indices by precision measure on four networks including Karate, CE, PB and Odlis. The detailed accuracy results can be found in Table S1 and Table S2.

Because FBM plus SBM and HRG are link predictors using global information of the network whereas classical link predictors such as CN, PA, AA, RA, and CAR-based methods using local information of the networks, it's not surprising that FBM performs slower than these methods. However, as for the running-time comparison results between these approaches shown in Table S3, beyond our expectation, we find that FBM approach performs even faster than CAR-based approaches and classical approaches on five networks. We think that the FBM possesses the good performance of scalability and is of a good tradeoff between prediction accuracy and computational efficiency.
